# The diagnostic performance of serum MUC5AC for cholangiocarcinoma: A systematic review and meta-analysis: Erratum

**DOI:** 10.1097/MD.0000000000006218

**Published:** 2017-02-24

In the article “The diagnostic performance of serum MUC5AC for cholangiocarcinoma: A systematic review and meta-analysis”,^[1]^ which appeared in Volume 95, Issue 24 of *Medicine*, the name of the authors’ affiliation should have appeared as Bayi Hospital Affiliated Nanjing University of Chinese Medicine.
